# Effect of Exogenous and Endogenous Ectoine on *Monascus* Development, Metabolism, and Pigment Stability

**DOI:** 10.3390/foods12173217

**Published:** 2023-08-26

**Authors:** Pengfei Gong, Ruoyu Shi, Jiali Tang, Jiaying Wang, Qiaoqiao Luo, Jia’ao Zhang, Xiaochun Ruan, Chengtao Wang, Wei Chen

**Affiliations:** 1 Key Laboratory of Geriatric Nutrition and Health, Ministry of Education, Beijing Advanced Innovation Center for Food Nutrition and Human Health, Beijing Engineering and Technology Research Center of Food Additives, School of Food and Health, Beijing Technology and Business University, Beijing 100048, China; 2130021003@st.btbu.edu.cn (P.G.); shiruoy@126.com (R.S.); 2230202138@st.btbu.cn (J.T.); 2230202145@st.btbu.edu.cn (J.W.); luoqiaoqiao7469@163.com (Q.L.); 13621013837@189.com (J.Z.); wangchengtao@th.btbu.edu.cn (C.W.); 2 Yanjin Biotechnology (Beijing) Co., Ltd., Beijing 102300, China; sino_bitech@163.com

**Keywords:** *Monascus purpureus*, ectoine, development, bioactive compounds, secondary metabolism

## Abstract

*Monascus*, a key player in fermented food production, is known for generating *Monascus* pigments (MPs) and monacolin K (MK), possessing bioactive properties. However, the limited stability of MPs and mycotoxin citrinin (CTN) constrain the *Monascus* industry. Extremolytes like ectoine, derived from bacteria, exhibit cytoprotective potential. Here, we investigated the impact of ectoine on *Monascus purpureus* ATCC 16365, emphasizing development and secondary metabolism. Exogenous 5 mM ectoine supplementation substantially increased the yields of MPs and MK (105%–150%) and reduced CTN production. Ectoine influenced mycelial growth, spore development, and gene expression in *Monascus*. Remarkably, ectoine biosynthesis was achieved in *Monascus*, showing comparable effects to exogenous addition. Notably, endogenous ectoine effectively enhanced the stability of MPs under diverse stress conditions. Our findings propose an innovative strategy for augmenting the production and stability of bioactive compounds while reducing CTN levels, advancing the *Monascus* industry.

## 1. Introduction

*Monascus* is a commonly found fungal species used in traditional Asian food production [1]. The species synthesizes secondary metabolites including *Monascus* pigments, monacolin K (MK), and citrinin (CTN) [2,3]. MPs are categorized into orange, yellow, and red pigments, and are utilized in a variety of industries including food and medicine [4,5]. Despite their widespread use, the production and stability of MPs are currently unable to meet the global market demands. Several strategies have been employed to improve MP production and stability such as cost-effective fermentation using low-cost agricultural by-products, small molecule supplementation, and mutagenesis for the selection of high-yield strains [6,7,8,9]. In recent studies, Mahmoud et al. [10] utilized mixed carbon sources to produce MPs and observed that the addition of antioxidants such as citric, ascorbic, and salicylic acids improved the light stability of MPs. Ali et al. [11] investigated the effect of sodium caseinate (SC) on the thermal stability of MPs under acidic conditions, finding that MP–SC complexes inhibit MP aggregation and enhance MP solubility and stability. Furthermore, Huang et al. [8] developed a novel integrated fermentation system comprising sodium starch, octenyl succinate, and Triton X-100, which increased the yield of extracellular red pigments in *Monascus purpureus* GDMCC 61166, thus offering an effective approach to augment MP biosynthesis and secretion. However, challenges such as cumbersome operation and raw material instability as well as the emergence of mutant strains remain significant obstacles in the application of this system.

Ectoine, a natural cytoprotective and stabilizing agent, was first discovered in *Ectothiorhodospira halochloris* by Schuh in 1985 [12]. With its ability to resist high temperature and osmotic pressure, ectoine has gained significant commercial value in various industries including biopharmaceuticals, biotechnology, and fine chemicals [13]. Additionally, ectoine has been shown to exhibit anti-aging and cytoprotective effects, and may have potential therapeutic applications for diseases such as Alzheimer’s disease [14,15]. Ectoine biosynthesis occurs through five sequential enzymatic reactions from L-aspartate, and its production is often triggered by increased environmental salinity [16]. Many halophilic and salt-tolerant bacteria produce ectoine, with the intracellular ectoine concentration being influenced by the NaCl content of the medium [17]. Current studies have reported the use of “bacterial milking” and synthetic biology for ectoine production, which includes the successful construction of metabolically engineered strains of *Escherichia coli* and *Corynebacterium glutamicum* for effective ectoine production using simple and inexpensive feedstock [18,19,20]. However, the potential for using *Monascus* as a host for ectoine production and the effect of ectoine on *Monascus* development and MP production and stability remains unexplored.

This study aimed to establish a new *Monascus* cell factory for the production of ectoine and assess the impact of ectoine on *Monascus* development, metabolism, and MP stability. The effects of exogenous ectoine on *Monascus* development and secondary metabolism were analyzed, and the *ectABC* cluster was heterologously expressed in *M. purpureus* ATCC 16365 to enable endogenous ectoine biosynthesis in *Monascus*. We hypothesized that both exogenous ectoine addition and endogenous ectoine production would enhance *Monascus* development and metabolism. Finally, we investigated whether endogenous ectoine production could improve the stability of MPs under different conditions such as light, thermal, and pH, thereby promoting the growth of the *Monascus* industry.

## 2. Materials and Methods

### 2.1. General

DNA polymerase mix kits were obtained from YEASEN Biotechnology Co., Ltd. (Shanghai, China). The Gibson assembly kit was acquired from GeneralBio Co., Ltd. (Chuzhou, China). Various chemicals including ectoine, L-aspartate, MK, CTN, ampicillin, hygromycin B, snail enzyme, cellulase, and lysozyme were purchased from Sigma-Aldrich Co., Ltd. (St. Louis, MO, USA). The following items were procured from Yanjin Biotechnology (Beijing) Co., Ltd. (Beijing, China): *E. coli* DH-5α, Polysaccharide Polyphenol Plant Total RNA Extraction Kit (Tiangen, Beijing, China), FastQuant RT Kit (with gDNase) (Tiangen, Beijing, China), and the SuperReal PreMix Plus (SYBR Green) Kit (Tiangen, Beijing, China).

### 2.2. Strains, Media, and Cultural Conditions

The *M. purpureus* strain ATCC 16365 (CGMCC 3.4446) was obtained from the China General Microbiological Culture Collection Center (CGMCC) and used as the wild-type (WT) control strain. Mycelium collection was performed by maintaining strains on potato dextrose agar (PDA) medium at 30 °C. For protoplast regeneration and transformation resistance screening, PDA supplemented with 1.2 M sorbitol and 20 µg/mL hygromycin B was used. Ampicillin (100 µg/mL) and 5 mM L-aspartate were added when necessary. Fresh spores (10^6^/mL) were inoculated into 50 mL of PDB medium and continuously agitated at 120 rpm and 28 °C. Morphological observations were carried out after 7 days of growth. *E. coli* DH-5α, cultured in LB medium, served as the host for conventional plasmid subcloning.

### 2.3. Construction and Validation of an Ectoine-Producing Monascus Cell Factory

The plasmids and primers used in this study are listed in Tables S1 and S2. The *ectABC* gene cluster from *Halomonas elongata* was synthesized by GeneralBio (Chuzhou, China) after codon optimization (Table S3). Primers were used to amplify the pathway fragments. The vector fragment was amplified using plasmid pBARGPE1-*hygro* (Miaoling, Wuhan, China) as the template, with primers vector-F/R. The purified gene fragments *ectA*, *ectB*, and *ectC* were assembled with the vector fragment using the Hieff Clone Plus Multi One Step Cloning Kit (YEASEN, Shanghai, China), resulting in plasmid pBARGPE-*hygro*-*ectABC* (P1), pBARGPE-*hygro*-*ectACB* (P2), pBARGPE-*hygro*-*ectBAC* (P3), pBARGPE-*hygro*-*ectBCA* (P4), pBARGPE-*hygro*-*ectCAB* (P5), and pBARGPE-*hygro*-*ectCBA* (P6). The three genes in different orders were organized into an operon expressed from the respective plasmids.

The construction of the *M. purpureus* ectoine-producing strain (MppECT) was performed as described by Shi et al. [21]. The pBARGPE-*hygro*-*ectABC* plasmid was incubated with 100 µL of *M. purpureus* ATCC 16365 WT strain protoplasts to construct the ectoine-producing strain (MppECT). The expression of the *ectABC* gene cluster was verified by diagnostic PCR. The transformant colonies were used as templates of diagnostic PCR.

### 2.4. Protoplasm System Preparation and Transformation

The spore suspension of the WT strain was prepared by adding sterilized water to the PDA medium. The resulting suspension was then applied (200 μL) onto cellophane-covered PDA medium and incubated at 37 °C for 36–40 h. The mycelium was subsequently harvested using a glass rod and transferred to nylon cloth, followed by washing with 0.5 M MgSO_4_ (50 mL). The mycelium was then transferred to an enzyme solution (20 mL of 0.5 M MgSO_4_ containing 0.2 g snail enzyme, 0.02 g cellulase, and 0.06 g lysozyme) that had been filtered and sterilized. The mycelium underwent enzymatic decomposition at 30 °C for 2.5–3 h at 80 rpm. The resulting lysate was filtered through nylon cloth into sterilized bottles, divided into centrifuge tubes, and centrifuged at 4 °C for 10 min at 7000 rpm. The supernatant was removed, and the pellet was washed twice with pre-cooled sorbitol solution, followed by a single wash with electric shock solution. Protoplasts were discarded by centrifugation under the same conditions. Finally, electric shock solution (220 μL) was added and mixed thoroughly. A mixture of 70 μL of cells and 10 μL of plasmid was transferred to a pre-cooled electroporation cuvette and subjected to transformation (220 V, 400 Ω, 25 uF). Subsequently, 900 μL of resuscitation medium (50 mL deionized water containing 1.75 g PDB) was added, and the mixture was incubated on ice for 10 min, followed by incubation at 30 °C for 2 h at 100 rpm. Conversion solution (200 μL) was then applied onto PDA medium containing various concentrations of hygromycin and incubated at 37 °C for 5–7 days.

### 2.5. Overexpression Strain Selection and Validation

Following the transformation, a selection of transformants was made by cultivating them on media containing different concentrations of hygromycin (0 μg/mL, 5 μg/mL, 10 μg/mL, and 15 μg/mL). The mycelia from the selected monoclonal transformants were subjected to boiling with a specific amount of deionized water to disrupt the cell wall. The resulting lysate served as the template for verification experiments using PCR. The PCR conditions involved an initial denaturation step at 94 °C for 90 s, followed by 30 cycles of denaturation at 94 °C for 20 s, annealing at 52 °C for 20 s, and extension at 72 °C for 5 min. Positive transformants were identified through this screening process and subsequently subcultured for further experimental procedures.

### 2.6. Condition of Culture

To prepare the spore suspension, a specific amount of sterilized water was introduced to the PDA medium containing *M. purpureus* strains. The resulting suspension was then added to a sterilized seed medium composed of 30 g/L glucose, 15 g/L soybean meal, 10 g/L peptone, 70 mL/L glycerol, 2 g/L KH_2_PO_4_, 2 g/L NaNO_3_, and 1 g/L MgSO_4_·7H_2_O at a volume of 5% relative to the seed culture medium. The mixture was incubated at 30 °C for 48 h with agitation at 200 rpm. Afterward, the seed culture medium was transferred to a sterilized fermentation medium consisting of 20 g/L rice meal, 10 g/L peptone, 90 mL/L glycerol, 2 g/L KH_2_PO_4_, and 5 g/L NaNO_3_ at a volume of 10% relative to the fermentation medium. The fermentation process commenced at 30 °C for 2 days with agitation at 200 rpm, followed by a subsequent incubation at 25 °C for 13 days with agitation at 200 rpm. On the second day of the fermentation process, different concentrations of ectoine were added to the fermentation medium to determine the optimal ectoine concentration.

### 2.7. Determination of Biomass, MPs, MK, and CTN

The analysis methods for the *M. purpureus* strains’ biomass and pigments were modified based on the procedure described by Shi et al. [22]. To determine the biomass concentration, the mycelia present in the fermentation broth were washed three times with distilled water to remove any adhering medium components. The washed mycelia were then filtered and collected. Next, the collected mycelia were dried at 60 °C until a constant weight was achieved. The weight obtained after drying represents the dry weight of the mycelia. The biomass concentration is expressed as the mycelial dry weight per unit volume of the fermentation broth and is typically reported in grams per liter (g/L). This measurement provides an indication of the amount of mycelia present in the fermentation broth. A 1 mL sample of the fermentation broth was combined with 3 mL of a 70% ethanol solution. The mixture was subjected to centrifugation at 5000 rpm for 10 min following a water bath treatment at 60 °C for 1 h. The resulting supernatant was appropriately diluted, and the absorbance values at wavelengths of 505 nm, 448 nm, and 410 nm were measured using a UV spectrophotometer. The obtained results were then calculated as optical density (OD) units multiplied by the corresponding dilution factor. The yellow pigment color value (U/mL) was determined by multiplying the OD value at 410 nm by the dilution factor. Similarly, the orange pigment color value (U/mL) was calculated by multiplying the OD value at 448 nm by the dilution factor, while the red pigment color value (U/mL) was determined by multiplying the OD value at 505 nm by the dilution factor.

To extract MK, a 1 mL sample of the fermentation broth was mixed with a 75% methanol solution (3 mL) and subjected to ultrasound treatment for 30 min. The mixture was then left to stand overnight and subsequently filtered using a 0.22 µm filter membrane. The detection of MK was performed using high-performance liquid chromatography (HPLC), and the content of MK was calculated based on a standard curve. The HPLC analysis was carried out using an Inertsil ODS-3 C_18_ column (150 mm × 4.6 mm × 5 μm). The mobile phase consisted of a mixture of 0.1% H_3_PO_4_ and methanol (in a ratio of 1:3). The flow rate was set at 1 mL/min, and detection was performed using an ultraviolet detector at a wavelength of 237 nm. The injection volume for each sample was 10 μL, and the analysis was conducted at a temperature of 30 °C.

For the extraction of CTN, the method described by Shi et al. [21] was employed. The CTN concentration in the fermentation broth was determined using ultra-performance liquid chromatography (UPLC) with an Agilent 1290 system (Waldbronn, Germany). The mobile phase consisted of solvent A (0.1% formic acid aqueous solution) and solvent B (acetonitrile) in a ratio of 0.1% formic acid to acetonitrile (1:1, *v*/*v*). The flow rate was set at 1 mL/min, and the injection volume was 2 µL. Detection was performed using a fluorescence detector at the excitation wavelength of 330 nm and the emission wavelength of 500 nm, and the column temperature was maintained at 30 °C.

### 2.8. Microscopy Analysis of Monascus Mycelia and Spore

For the observation of spore and mycelia morphology, SEM was employed. An appropriate volume of the *M. purpureus* fermentation liquid was centrifuged at 12,000 rpm for 5 min, and the supernatant was discarded. The mycelia were fixed with a suitable amount of 2.5% glutaraldehyde solution under conditions avoiding exposure to light. Subsequently, the mycelia were subjected to gradient elution using ethanol solutions with different concentrations (50%, 70%, 85%, 95%, and 100%). After standing for 10 min, the supernatant was removed by centrifugation at 12,000 rpm for 5 min. Finally, the mycelia samples were prepared through vacuum freeze-drying and gold spraying (2 min) for SEM imaging.

### 2.9. Real-Time Quantitative PCR (RT-qPCR) Analysis

The fermentation broth of the *M. purpureus* strains was subjected to centrifugation at 12,000 rpm for 5 min. The resulting supernatant was discarded, and the mycelia were washed four times with sterilized water. Total mycelial RNA was extracted using the RNAprep Pure Plant Kit, and first-strand cDNA synthesis was performed using the FastQuant RT Kit (with gDNase). RT-qPCR was employed to monitor the gene expression levels of various pathways involved in MK synthesis (*mokA*, *mokB*, *mokC*, *mokD*, *mokE*, *mokF*, *mokG*, *mokH*, and *mokI*), MP synthesis (*mppA*, *mppB*, *mppC*, *mppD*, *mppE*, *mppG*, *mpp7*, *mppR1*, *mppR2*, *pksPT*, *MpFasA2*, and *MpFasB2*), CTN synthesis (*pksCT*, *ctnA*, *ctnB*, *ctnR*, *orf1*, and *orf7*), the global regulator gene *laeA*, morphology regulatory genes *brlA*, *wetA*, *veA*, *velB*, *velC*, *vosA*, and reference genes *GADPH*. RT-qPCR was performed using a CFX96 Real-Time PCR Detection System. The amplification conditions for RT-qPCR were as follows: an initial denaturation step at 95 °C for 15 min, followed by 40 cycles of denaturation at 95 °C for 10 s, annealing at 52 °C for 20 s, and extension at 72 °C for 30 s. The relative expression levels were calculated using the 2^−ΔΔCt^ method, where ΔCt represents the difference in cycle threshold values between the target genes and the reference genes.

### 2.10. Stability Analysis of MPs

Light stability: The evaluation of light stability was conducted following the methodology described by Terán Hilares et al. [23], with slight modifications. During fermentation, LED lights emitting specific wavelengths were employed to illuminate each Erlenmeyer flask. The red pigment production was assessed using LED lights with wavelengths ranging from 620 to 630 nm, green pigment with wavelengths from 515 to 535 nm, and blue pigment with wavelengths from 450 to 470 nm. The photon flux density utilized was 250 µmol·m^−2^·s^−1^, and the distance between the Erlenmeyer flask and the LED light source was maintained at approximately 6 cm. For comparison, control experiments were carried out under dark conditions (Erlenmeyer flask completely covered). All experiments were conducted in triplicate at a temperature of 30 °C and a rotational speed of 200 rpm for a duration of 12 days. Periodic sampling was performed to monitor substrate consumption, cell growth, and MP production.

Thermal and pH stability: The evaluation of thermal and pH stability of the red pigment followed the methodology described by Chen et al. [24], with slight modifications. The thermal stability was assessed by subjecting the red pigments produced in submerged fermentation to various temperatures (50, 70, and 90 °C) and pH values (3, 4, 5, 6, 7, and 8). The pH of the solution was adjusted using 0.1 M acetic acid or sodium hydroxide aqueous solutions. To determine the thermal stability, the thermal treatment of the red pigment solution (2 mL) to each experimental condition was performed in sealed 5-mL test tubes, which were incubated in a water bath. The absorbance (A) of the red pigment solutions was measured at the maximum wavelength of 505 nm at different treatment times (t) under the specified temperatures. The pigment degradation kinetics were modeled using first-order reaction kinetics, as shown in Equation (1), where A_t_/A_0_ represents the ratio of absorbance at time t to the initial absorbance, and D_k_ is the deactivation rate constant estimated from the slope of the semi-logarithmic plot of A_t_/A_0_ versus t. The pH stability was evaluated by subjecting the red pigment solutions to different pH levels, and the absorbance at 505 nm was measured. The same first-order reaction kinetics model was used to analyze the degradation kinetics. The half-life time (t_1/2_) of the red pigment, representing the time required for the pigment to degrade by half, was calculated using Equation (2) by using the deactivation rate constant (D_k_). The temperature dependence of the red pigment produced by the WT and MppECT strains were assessed using the Arrhenius model, represented by Equations (3) and (4). In these equations, D_0_ represents the pre-exponential factor, E_a_ represents the activation energy in kcal·mol^−1^, R is the universal gas constant (1.987 cal·mol^−1^·K^−1^), and T represents the absolute temperature in Kelvin.

Ln A_t_/A_0_ = −D_k_t (1)

t_1/2_ = Ln 2/D_k_ (2)

D_k_t = D_0_e^−Ea/RT^ (3)

LnD_k_ = LnD_0_ − E_a_/RT (4)

### 2.11. Statistical Analysis

Each experiment was conducted with a minimum of three parallel replicates. Statistical analyses were performed using one-way analysis of variance (ANOVA) with the aid of GraphPad Prism 9.0 software. The numerical data were presented as mean ± standard deviation (SD), and *p*-values less than 0.05 and 0.01 were considered statistically significant.

## 3. Results

### 3.1. Effect of Exogenous Ectoine Addition on Monascus Growth and Metabolite Production

**Figure 1:**
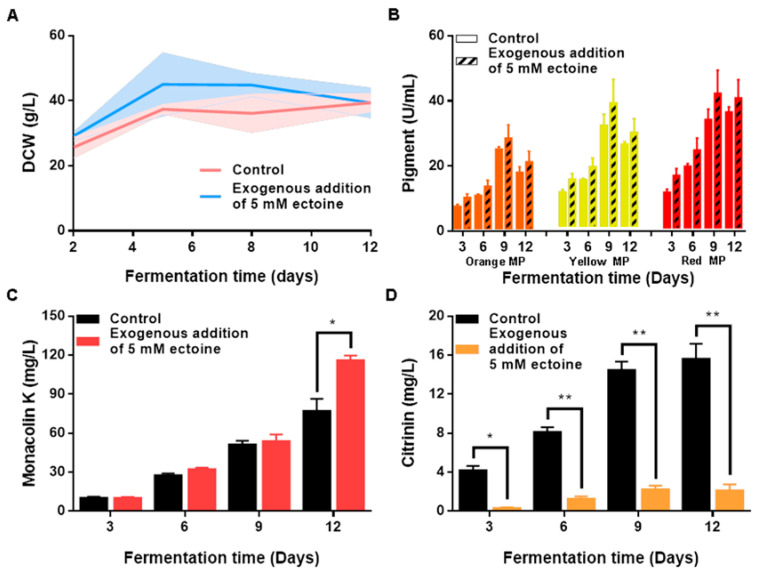
Effects of exogenous ectoine on *Monascus* growth and secondary metabolism. (**A**) Dry cell weight. (**B**) Production of *Monascus* pigments. (**C**) Production of Monacolin K. (**D**) Production of citrinin. * *p* < 0.05 and ** *p* < 0.01.

In this study, we investigated the protective effects of ectoine on the filamentous fungus *Monascus*. Specifically, we examined the effects of the exogenous addition of 5 mM ectoine on the culture dry cell weight (DCW) as well as on the yields of three major MPs, MK, and CTN during the fermentation process (Figure 1). Our results indicate that ectoine is an effective growth stimulant for *Monascus*, as evidenced by the acceleration in the growth rates observed in the presence of 5 mM ectoine (Figure 1A). Furthermore, we found that the addition of ectoine increased the yields of all three MPs (orange, yellow, and red pigments) at different time points during the incubation period, with the greatest increase in yields observed on day 9 (Figure 1B). The maximum yields achieved for the orange, yellow, and red pigments were 138%, 136%, and 146% higher than the control group, respectively. Additionally, we observed that ectoine treatment stimulated the production of MK, with the maximum yield reaching 150% higher than that of the control group on day 12 (Figure 1C). However, the production of CTN, a potent nephrotoxic and hepatotoxic mycotoxin that hinders the development of the *Monascus* industry, was significantly reduced by the addition of 5 mM ectoine (Figure 1D).

### 3.2. Effect of Exogenous Ectoine Addition on Monascus Morphology and Gene Transcription

**Figure 2:**
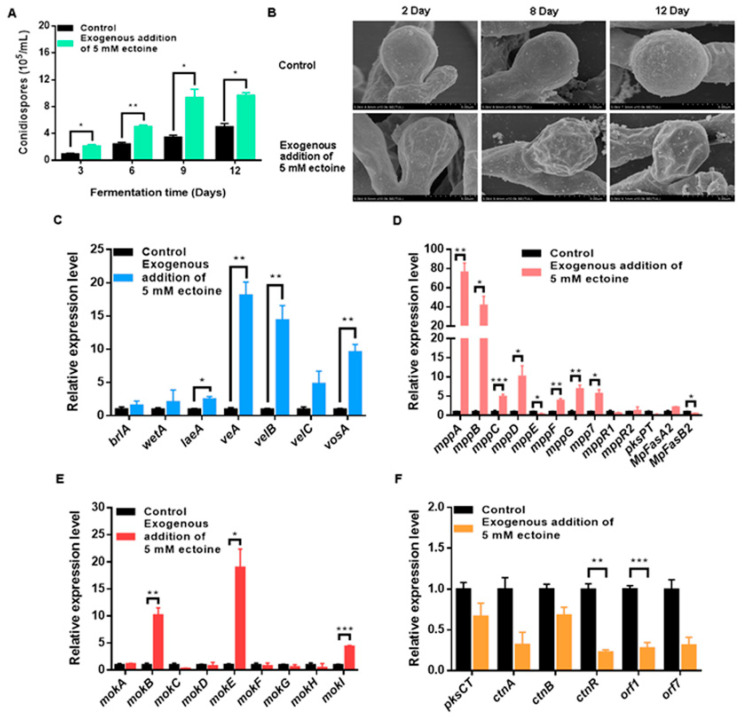
Effects of exogenous ectoine on *Monascus* development and gene expression. (**A**) Number of conidispores. (**B**) SEM observation (at 10,000× magnification). (**C**) Transcriptional expression level of genes relevant to development. (**D**) Transcriptional expression level of genes relevant to *Monascus* pigment biosynthesis. (**E**) Transcriptional expression level of genes relevant to Monacolin K biosynthesis. (**F**) Transcriptional expression level of genes relevant to citrinin biosynthesis. * *p* < 0.05, ** *p* < 0.01. and *** *p* < 0.001.

In the current study, we investigated the effect of ectoine supplementation on *Monascus* growth and morphology by inoculating *M. purpureus* ATCC 16365 conidia onto solid PDA with or without 5 mM ectoine at 30 °C for 12 days. Our results demonstrated that the ectoine-supplemented group produced a greater number of conidiospores compared with the control group (Figure 2A). Scanning electron microscopy (SEM) further revealed that spores in the ectoine-supplemented group exhibited increased pitting and folding, and that the group exhibited smoother and fuller mycelia and numerous closed ascospores (Figure 2B).

Our investigation into the regulatory genes involved in *Monascus* growth and conidial development revealed that ectoine treatment resulted in significant upregulation of several key regulators. Specifically, we found that ectoine treatment upregulated the expression of *brlA*, *wetA*, *laeA*, *veA*, *velB*, *velC*, and *vosA* by 1.54, 2.10, 2.52, 18.11, 14.37, 4.84, and 9.58-fold, respectively (Figure 2C). The expression of key genes involved in *Monascus* conidial development and biosynthesis of the main secondary metabolites were also analyzed. As shown in Figure 2D, the results indicate that ectoine can considerably upregulate the expression of several key genes involved in MP biosynthesis including *MpPKS5*, *MpFasA2*, *mppA*–*mppG*, *mpp7*, *mppR1*, and *mppR2*. These genes encode polyketide synthases, fatty acid synthases, acetyltransferases, oxidoreductases, and regulatory proteins that play crucial roles in MP production.

Furthermore, the present study also investigated the effect of ectoine on the expression of key genes involved in MPs and MK biosynthesis as well as the biosynthesis of CTN. The results indicated that ectoine supplementation upregulated the expression of *mokA*–*mokI*, which encode key enzymes involved in MK biosynthesis, exhibiting a 1.18–18.42-fold increase compared with the control group. However, the fold-change of the *mok* gene cluster expression was lower than that of the *mpp* gene cluster, consistent with the observed final yields of MPs and MK (Figure 2E). In contrast, ectoine treatment downregulated the expression of key genes involved in CTN biosynthesis including *pksCT*, *ctnA*, *ctnB*, *ctnC*, *ctnR*, *orf1*, and *orf7*, as shown in Figure 2F.

### 3.3. Construction and Validation of an Ectoine-Producing Monascus Cell Factory

**Figure 3:**
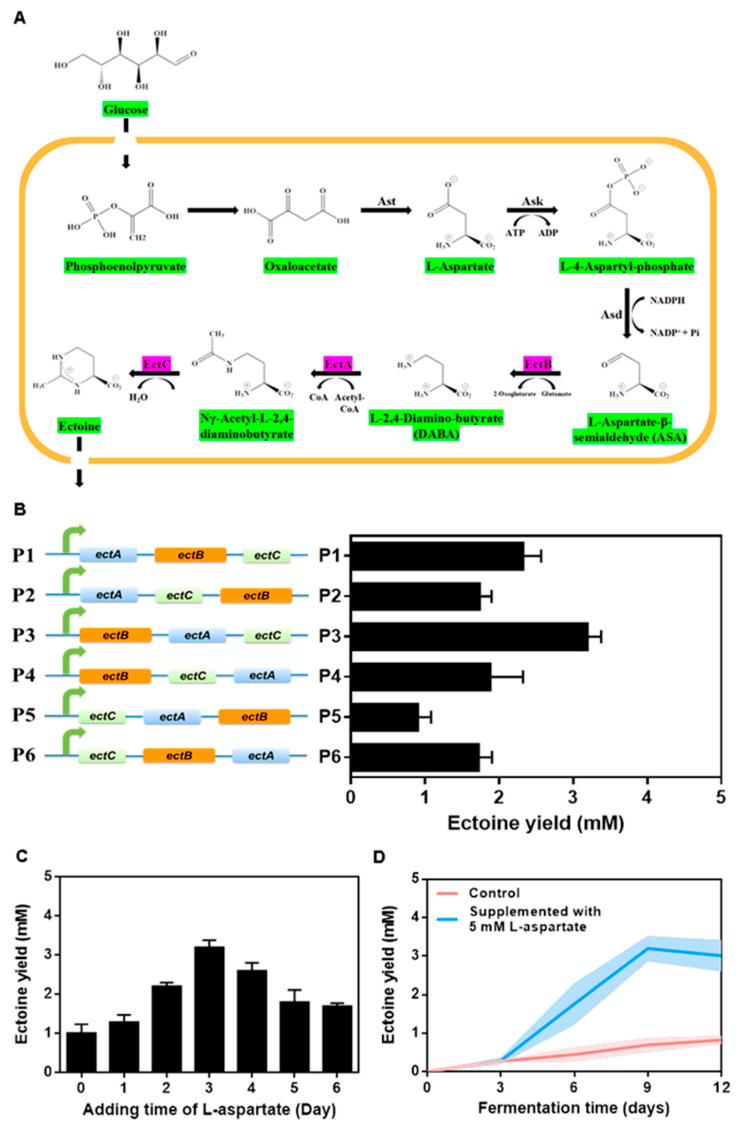
Construction and characterization of ectoine-producing *Monascus* cell factory. (**A**) Ectoine biosynthetic pathway. (**B**) Optimization of the *ectABC* operon. (**C**) Optimization of L-aspartate addition. (**D**) Production of ectoine.

In the current study, the ectoine biosynthetic pathway was expressed in *M. purpureus* ATCC 16365 to establish an ectoine-producing *Monascus* cell factory. The heterologous pathway consists of three genes, *ectA*, *ectB*, and *ectC*, which encode L-diaminobutyric acid acetyl transferase, L-2,4-diaminobutyric acid (DABA) transaminase, and ectoine synthase, respectively (Figure 3A). The plasmid pBARGPE-*hygro*-*ectBAC* (Figure S1), carrying the heterologous ectoine biosynthetic pathway, was verified through diagnostic PCR and fermentation, with bands for the operon detected on the gels (Figure S2). To optimize the ectoine biosynthetic efficiency, we optimized the order of the three genes in the operon. L-Aspartate was used as a substrate for the pathway, and the operon in plasmid P3 produced the highest amount of ectoine (Figure 3B). Optimization of the addition timepoint of L-aspartate indicated that adding L-aspartate on day 3 maximized the ectoine yield (Figure 3C). Analysis of the ectoine yield during the fermentation process revealed the highest ectoine yield on day 9 with 5 mM L-aspartate supplementation, which was 459% higher than that of the control group (Figure 3D).

### 3.4. Effect of Endogenous Ectoine Biosynthesis on the Development and Secondary Metabolism of Monascus

**Figure 4:**
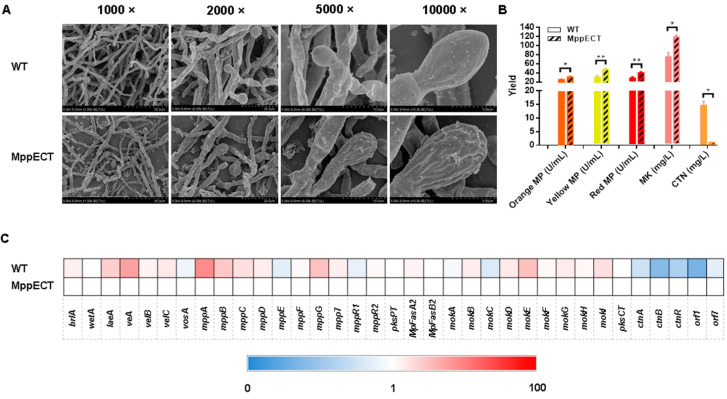
Effects of endogenous ectoine on *Monascus* development and metabolism. (**A**) SEM observation (at 1000×, 2000×, 5000× and 10,000× magnifications). (**B**) Production of *Monascus* pigment (U/mL), Monacolin K (mg/L) and citrinin (mg/L). (**C**) Heatmap of the transcriptional expression level of genes relevant to development and metabolism. * *p* < 0.05 and ** *p* < 0.01.

Investigation into the effects of endogenous ectoine on *Monascus* development and secondary metabolism indicated that similar to exogenous ectoine addition, the production of endogenous ectoine increased the DCW of the *Monascus* culture (Figure S3). Additionally, the ectoine-producing group displayed accelerated mycelium growth, increased mycelium density and spore number, and reduced roughness of the spore surface (Figure 4A and Figure S4). Notably, the MP and MK yields in the ectoine-producing group were significantly higher than those in the control group, increasing by 121% (orange), 154% (yellow), 140% (red), and 159% (MK) (Figure 4B). Furthermore, the CTN yield in the ectoine-producing group was 94% lower than that of the control group (Figure 4B).

In order to elucidate the underlying mechanisms behind the observed effects of ectoine on *Monascus*, we conducted a transcriptional analysis to investigate the expression levels of genes involved in both development and secondary metabolism in *Monascus* (Figure 4C). Consistent with the effects of exogenous ectoine addition, endogenous ectoine production was found to upregulate the expression of the *mpp* and *mok* gene clusters, as shown in the heatmap (Figure 4C), while significantly downregulating the expression of genes associated with CTN biosynthesis.

### 3.5. Protective Effect of Endogenous Ectoine Biosynthesis on the Stability of Red Pigment

**Figure 5:**
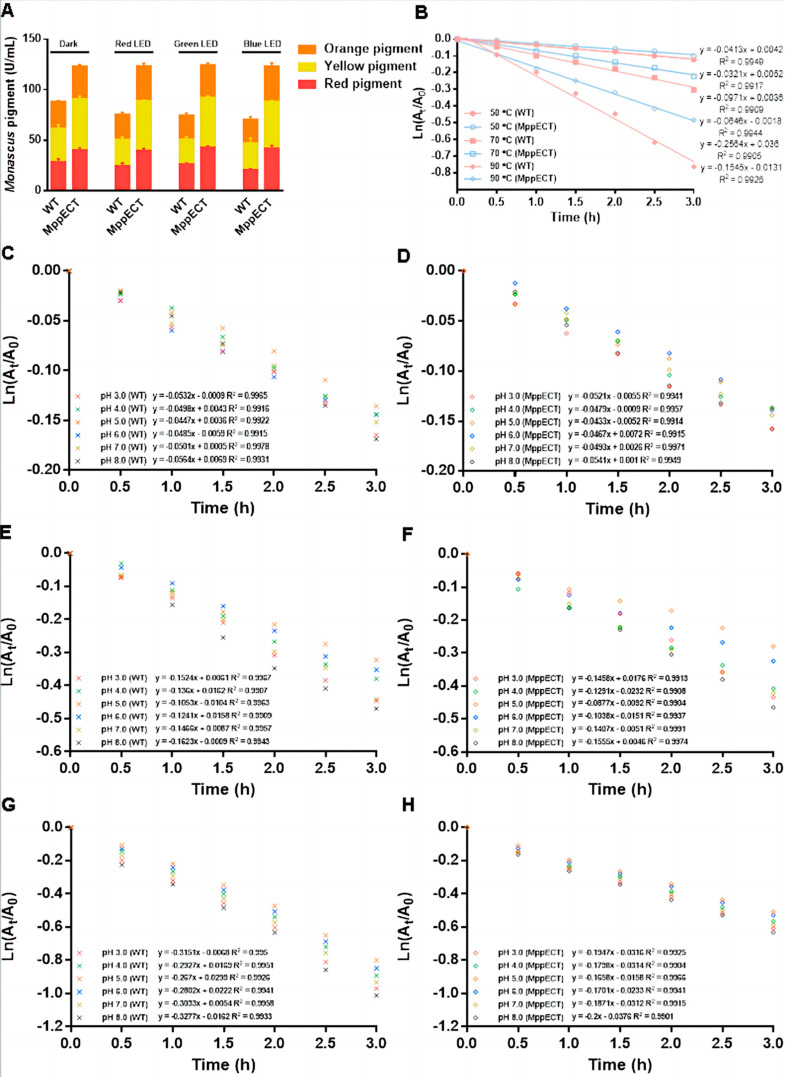
Effects of endogenous ectoine on *Monascus* pigment stability. (**A**) Thermal stability of the red pigment. (**B**) Thermal stability of the red pigment. (**C**) Thermal degradation kinetics of the red pigment produced by the WT strain at various pH and 50 °C. (**D**) Thermal degradation kinetics of the red pigment produced by the MppECT strain at various pH and 50 °C. (**E**) Thermal degradation kinetics of the red pigment produced by the WT strain at various pH and 70 °C. (**F**) Thermal degradation kinetics of the red pigment produced by the MppECT strain at various pH and 70 °C. (**G**) Thermal degradation kinetics of the red pigment produced by the WT strain at various pH and 90 °C. (**H**) Thermal degradation kinetics of the red pigment produced by the MppECT strain at various pH and 90 °C.

The effect of ectoine on MPs was further investigated by assessing the protective effect of endogenous ectoine on the stability of MPs produced during *Monascus* fermentation. As shown in Figure 5A, the effect of different LED wavelengths on MP production on day 9 was evaluated, and it was found that MP synthesis in the control group was hindered by LED exposure, with the lowest MP production observed during fermentation under blue LED light. In the ectoine-producing group, the MPs exhibited enhanced light stability and the destructive effect of LED light on MP production was attenuated. The lowest MP production was promoted in fermentation with the incidence of blue LED light. Of the different MPs, the yellow pigment exhibited the greatest photostability, followed by the red pigment, whereas the orange pigment was the least photostable.

The thermal degradation behavior of red pigment produced in the control and ectoine-producing groups at pH 5.5 was evaluated using first-order kinetic models, and the results are shown in Figure 5B. Both groups exhibited accelerated red pigment degradation with increasing temperature and time. However, endogenous ectoine provided excellent protection against pigment degradation under high-temperature exposure. The degradation profiles were fitted to a first-order kinetic model with a high regression coefficient (R^2^ > 0.99) for both groups.

The effect of endogenous ectoine production on pH- and the thermostability of MPs was evaluated by culturing control and ectoine-producing groups at different pH (3.0–8.0) and temperature (50, 70, and 90 °C) conditions (Figure 5C–H). The results, summarized in Table 1, showed that both groups exhibited increased thermal degradation constant (D_k_) and decreased half-life (t_1/2_) as the temperature increased from 50 to 90 °C at all pH levels studied. However, the ectoine-producing group showed a stronger protective effect for the red pigment, with lower fold-changes in D_k_ and t_1/2_ compared to the control group. At pH 5.0, the red pigment exhibited the highest t_1/2_ of 3.83 h (MppECT) and 3.80 h (WT) at 50 °C, which decreased to 2.49 h (MppECT) and 2.01 h (WT) at 90 °C. The t_1/2_ decreased further to 2.30 h (MppECT) and 1.81 h (WT) when the pH increased to 8.0, and to 2.33 h (MppECT) and 1.85 h (WT) when the pH decreased to 3.0. The ectoine-producing group exhibited lower D_k_ values compared to the control group, with the lowest D_k_ observed at 50 °C and pH 5.0. The activation energies (E_a_) of the red pigment produced in both groups were calculated for temperatures of 50 °C to 90 °C at each pH level studied.

## 4. Discussion

Ectoine is a compatible solute naturally present in bacterial cells that functions as a protectant against harsh conditions, thus promoting cell growth and metabolism. Prior research has documented the protective effects of ectoine in bacteria, animals, and humans [14]. Ectoine, as a compatible solute and osmoprotectant, can protect halophilic microorganisms from stress factors and also reduce the wrinkled appearance of the spore [25]. Our findings highlight the potential of ectoine as an effective growth-promoting agent and morphological modifier in *Monascus* culture. In recent years, the influence of culture conditions and external factors on *Monascus* morphology has been increasingly studied. Studies by Huang et al. [26] and Huang et al. [8] reported morphological changes in *M. purpureus* induced by microparticle addition during submerged fermentation. In this study, the effects of ectoine on the filamentous fungus *Monascus* were investigated and the findings suggest that ectoine treatment may serve as an effective alternative for controlling CTN production in *Monascus*. Because the main polyketone secondary metabolites of *Monascus* originate from malonyl-CoA and acetyl-CoA [27], we proposed that the increased production of MPs and MK resulting from the addition of ectoine may account for the observed reduction in CTN production. Our results also demonstrated that the ectoine considerably influenced the number of spores as well as the morphology of mycelia and spores.

Previous research has demonstrated that BrlA, AbaA, and WetA play critical roles in the regulation of conidiophore and bud formation in numerous filamentous fungi. However, Jia et al. [28] showed that the central regulatory model of conidiation is not applicable to *Monascus*, as the functional characterization of *brlA* and *wetA* in *Monascus ruber* M7 using gene knockout and overexpression revealed different regulatory mechanisms. Our study supports the notion that *brlA* and *wetA* expression levels are linked to morphological changes in *M. purpureus* ATCC 16365. However, the regulatory networks governing asexual development in filamentous fungi, especially in *Monascus*, require further study. In *Aspergillus*, VeA, VelB, VelC, and VosA play important roles in regulating hyphal organization and the alternation between primary and secondary metabolism, while LaeA contributes to this regulation and production [29]. Our results suggest that ectoine plays a role in cellular differentiation in *Monascus*, as we found that ectoine treatment significantly influences the mycelial and spore development of *Monascus* and upregulates the expression of key regulatory genes.

In addition to changes in morphology, the addition of compounds or microparticles can influence gene expression relevant to secondary metabolism, as demonstrated by several studies. Zhang et al. [30] reported that glutamic acid can enhance the expression of *mokC* and *mokG*, leading to an increase in MK production. Similarly, Yin et al. [31] investigated the effects of 20 free amino acids on gene transcription and observed that histidine and methionine can regulate MP biosynthesis. Huang et al. [8] also showed that treatment with microparticles significantly upregulated the expression of key genes involved in MP biosynthesis, namely *PksPT*, *PksCT*, *MppB*, *MpFasA2*, *MpFasB2*, and *MpPKS5*. In the present study, the upregulation of these genes (*MpPKS5*, *MpFasA2*, *mppA*–*mppG*, *mpp7*, *mppR1*, and *mppR2*) by ectoine treatment could increase the relative enzymatic activity and thus enhance MP biosynthesis. Additionally, the upregulation of *mppC*, *mpp7*, and *mppG* enhances the conversion of orange pigments to yellow and red pigments in *Monascus*. This finding sheds light on the regulatory mechanisms underlying pigment biosynthesis and provides a valuable means to manipulate and control the color output of *Monascus*. It unlocks the potential for customizing the color palette of *Monascus*, opening doors to innovation in various industries that rely on natural pigments. Interestingly, ectoine supplementation upregulated the expression of genes involved in MK biosynthesis, while downregulated those involved in CTN biosynthesis, suggesting that the increased biosynthesis of MPs and MKs may have resulted in the elimination of CTN biosynthesis, possibly due to competition for common biosynthetic precursors or limited metabolic capacity. In another study [32], rutin and its derivatives have been found to affect CTN production by *M. aurantiacus* Li AS3.4384 in liquid fermentation using different media types. Rutin and its derivatives may have inhibitory effects on citrinin production, potentially making them useful in reducing harmful metabolite formation. Additionally, the effects of exogenous ascorbic acid have been explored in *M. ruber* [33]. The addition of ascorbic acid impacts the yields of CTN and pigments as well as the antioxidant capacities and fatty acid composition. Ascorbic acid supplementation appears to have an influence on secondary metabolite production and antioxidant properties of the fermentation process. Furthermore, the addition of genistein to the fermentation process of *Monascus* species, as observed in one study [34], leads to reduced CTN production. This reduction is attributed to changes at the transcriptional level. Genistein has the potential to alter gene expression and subsequently impact secondary metabolite biosynthesis. Overall, these studies collectively suggest that exogenous compounds like ectoine, rutin, ascorbic acid, and genistein can have significant effects on the secondary metabolism of *Monascus* species. Their influence can range from inhibiting harmful metabolites to enhancing the antioxidant properties and impacting on gene expression related to secondary metabolite production. However, the specific mechanisms and outcomes might differ between compounds and species, so it is important to consider these variations when designing further research or applications.

The current study aimed to investigate the potential protective effect of endogenous ectoine production in *Monascus*. Ectoine is an osmoprotectant that has been shown to accumulate within cells without disturbing natural processes. Although ectoine production has been observed in engineered bacteria such as *E. coli* and *C. glutamicum*, its production in *Monascus* has yet to be reported [18,19]. In a previous study, an ectoine-responsive biosensor was designed and utilized to enhance ectoine biosynthesis in *E. col* [35]. In the current study, the ectoine biosynthetic pathway consisting of *ectA*, *ectB*, and *ectC* was expressed and optimized in *M. purpureus*, providing promising insights into the potential for endogenous ectoine production in *Monascus* as a protective mechanism for its morphology and metabolism. Despite the relatively low ectoine yield achieved by the engineered *Monascus* cell factory, the production of endogenous ectoine was found to have a promising cytoprotective effect, which is similar to exogenous ectoine treatment. Our results demonstrate that the production of endogenous ectoine is superior to exogenous ectoine addition in promoting *Monascus* development and metabolism. As an endogenous cytoprotective agent, intracellular ectoine stabilizes cells from the inside, which may be more direct and sensitive than extracellular ectoin [36]. Although the intracellular ectoine concentration was lower than that of exogenous ectoine, our findings indicate that intracellular ectoine exhibited a greater protective effect. Further optimization of the ectoine-producing *Monascus* cell factory will therefore facilitate the improved production of bioactive compounds.

The results of the transcriptional analysis of genes involved in both development and secondary metabolism in *Monascus* producing endogenous ectoine were consistent with the effects of exogenous ectoine addition. It was hypothesized that the intracellular accumulation of ectoine may have resulted in self-inhibition of the production of virulence factors [37]. However, the precise regulatory mechanisms underlying the inhibition of CTN biosynthesis by ectoine remain to be further investigated. It is interesting to note that endogenous ectoine production had a significant impact on the expression of genes related to both secondary metabolism and development in *Monascus*. The upregulation of *brlA*, *wetA*, and *laeA* suggests that endogenous ectoine might play a crucial role in asexual developmental processes [38], while the downregulation of genes associated with CTN biosynthesis indicates that endogenous ectoine can inhibit virulence factor production [39]. However, the fact that endogenous ectoine did not affect the expression of Velvet-family genes suggests that the regulation of asexual development in *Monascus* is more complex than previously thought, and different pathways might respond to different signals. Further investigation is needed to fully understand the mechanisms underlying the effects of endogenous ectoine on *Monascus* development and metabolism.

In the present study, endogenous ectoine biosynthesis was found to influence the yield and stability of MPs. The lowest MP production was promoted in fermentation with the incidence of blue LED light, which was also observed by previous studies [23,40]. Previous studies have used various methods to enhance MP photostability such as using non-ionic surfactants or antioxidants [41]. Herein, we report for the first time that ectoine can improve the photostability of MPs, and that endogenous ectoine production may be a promising way to improve MP stability. The thermal degradation behavior of red pigment demonstrates that endogenous ectoine plays a crucial role in enhancing MP stability under varying pH ranges and at high temperatures. Evaluation of the effect of endogenous ectoine on pH and the thermostability of the MPs showed results that were very similar to previous studies [23,24]. Further analysis showed that endogenous ectoine enhances the pH and thermostability of *Monascus* red pigment, with the E_a_ values being lower in the MppECT group compared to the WT group, indicating greater stability.

While acknowledging the advancements and studies that have been made in the realm of genetic manipulation and gene editing, it is apparent that *Monascus* species have not received the same level of attention in this field as extensively studied microorganisms like bacteria and yeast. This discrepancy can be attributed to several factors. One of these factors is the inherent complexity of genetic manipulation within Monascus species, which possess intricate growth and reproductive cycles. Additionally, the absence of well-established techniques for genetic engineering in *Monascus* has contributed to the relative dearth of progress in this area. Concerns pertaining to regulatory compliance and safety considerations surrounding genetic modifications in organisms intended for use in food and consumer goods have also played a role in shaping the trajectory of research. Furthermore, research priorities and funding allocations have influenced the rate of advancement in this field. However, it is essential to recognize that the research landscape is dynamic and subject to change over time. Recent years have seen rapid developments in gene editing tools and techniques, potentially altering the trajectory of research on *Monascus* species [21,42,43,44,45], underscoring the improved understanding and exploration of genetic manipulation in *Monascus*.

## 5. Conclusions

The present study reports the construction of a novel ectoine-producing *Monascus* cell factory and investigates the impact of ectoine on the growth, development, and secondary metabolism of *M. purpureus* ATCC 16365 as well as the light-, thermal-, and pH-stability of MPs. The results support the hypothesis that both the exogenous addition and endogenous production of ectoine significantly promote *Monascus* growth and development, enhance MP and MK production, reduce CTN biosynthesis, and significantly improve MP stability. Gene expression analysis revealed that ectoine regulates key genes involved in the morphological development and secondary metabolism of *Monascus*. Moreover, the study demonstrates that ectoine serves as a protective agent against the light, heat, and pH-induced degradation of MPs. Future research efforts should focus on improving ectoine yield and elucidating the impact of endogenous ectoine on the molecular structure and activity of bioactive food compounds. The findings of this study suggest that the heterologous biosynthesis of natural protectants is a practical and cost-effective strategy for enhancing the stability of bioactive food compounds and in promoting the development of the *Monascus* industry. In comparison to engineered strains of *E. coli* and *C. glutamicum*, genetically engineered *Monascus* offers several benefits including high tolerance to salt, acid, and ethanol as well as the ability to effectively utilize low-cost agricultural by-products, thereby making it an attractive candidate for the production of value-added compounds. However, research on the genetic manipulation in *Monascus* is currently lacking compared to bacteria and yeast, leading to a lack of gene-editing tools and *Monascus* chassis cells. Future studies should focus on the construction and standardization of additional genetically engineered *Monascus* strains as well as the development of gene-editing tools for this organism.

## Figures and Tables

**Table 1:** Thermal degradation characteristics of the red pigment from different temperature and pH conditions using *Monascus purpureus* ATCC 16365 strains.

pH	Temperature (°C)	Degradation Constant (D_k_, h^−l^)		Half Time (t_1/2_, h)		Activation Energy (E_a_, kJ mol^−1^)	
		WT	MppECT	WT	MppECT	WT	MppECT
	50	0.0532	0.0521	3.63	3.65		
3.0	70	0.1524	0.1458	2.57	2.62	10.40	7.76
	90	0.3151	0.1947	1.85	2.33		
	50	0.0498	0.0479	3.69	3.73		
4.0	70	0.136	0.1291	2.69	2.74	10.34	7.78
	90	0.2927	0.1798	1.92	2.41		
	50	0.0447	0.0433	3.80	3.83		
5.0	70	0.1053	0.0877	2.94	3.13	10.40	7.83
	90	0.267	0.1658	2.01	2.49		
	50	0.0485	0.0467	3.72	3.76		
6.0	70	0.1241	0.1038	2.78	2.96	10.23	7.56
	90	0.2802	0.1701	1.97	2.46		
	50	0.0501	0.0493	3.69	3.70		
7.0	70	0.1466	0.1407	2.61	2.65	10.52	7.85
	90	0.3033	0.1871	1.89	2.37		
	50	0.0564	0.0541	3.59	3.61		
8.0	70	0.1623	0.1555	2.51	2.55	10.29	7.70
	90	0.3277	0.2	1.81	2.30		

## Data Availability

The data used to support the findings of this study can be made available by the corresponding author upon request.

## Supplementary Materials

The following supporting information can be downloaded at: https://www.mdpi.com/article/10.3390/foods12173217/s1, Figure S1. Plasmid map of pBARGPE1-hygro-ectBAC; Figure S2. Diagnostic PCR of the expression of the ectBAC gene cluster. Lane 1: DL5000 DNA ladder. Lane 2: Blank. Lanes 3 and 4: ectBAC fragment (~2000 bp); Figure S3. Cell growth of the *Monascus purpureus* ATCC 16365 WT and MppECT strains; Figure S4. Spore number of *Monascus purpureus* ATCC 16365 WT and MppECT strains; Table S1. Plasmids used in this study; Table S2. Primers used in this study; Table S3. Sequence of gene cluster after codon optimization.
