# Supplementary material for: Effect of Exogenous and Endogenous Ectoine on Monascus Development, Metabolism, and Pigment Stability
Source: Foods. 2023 Aug 26;12(17):3217. doi: 10.3390/foods12173217 (PMC10486468; doi:10.3390/foods12173217)
Supplement: Supplementary file 1 [file foods-12-03217-s001.zip › foods-2565309-supplementary.pdf]

Supplementary Materials

Article

Effect of exogenous and endogenous ectoine on *Monascus* development, metabolism, and pigment stability

Pengfei Gong [1], Ruoyu Shi [1], Jiali Tang [1], Jiaying Wang [1], Qiaoqiao Luo [1], Jia'ao Zhang [1], Xiaochun Ruan [2], Chengtao Wang [1], and Wei Chen [1,*]

[1]Key Laboratory of Geriatric Nutrition and Health, Ministry of Education, Beijing Advanced Innovation Center for Food Nutrition and Human Health, Beijing Engineering and Technology Research Center of Food Additives, School of Food and Health, Beijing Technology and Business University, Beijing 100048, China; 2130021003@st.btbu.edu.cn (P.G.); shiruoy@126.com (R.S.); 2230202138@st.btbu.cn (J.T.); 2230202145@st.btbu.edu.cn (J.W.); luoqiaoqiao7469@163.com (Q.L.); 13621013837@189.com (J.Z.); wangchengtao@th.btbu.edu.cn (C.W.)

[2]Yanjin Biotechnology (Beijing) Co., Ltd., Beijing 102300, China; sino_bitech@163.com

* Correspondence: weichen@btbu.edu.cn

Tel.: +86-10-68984003

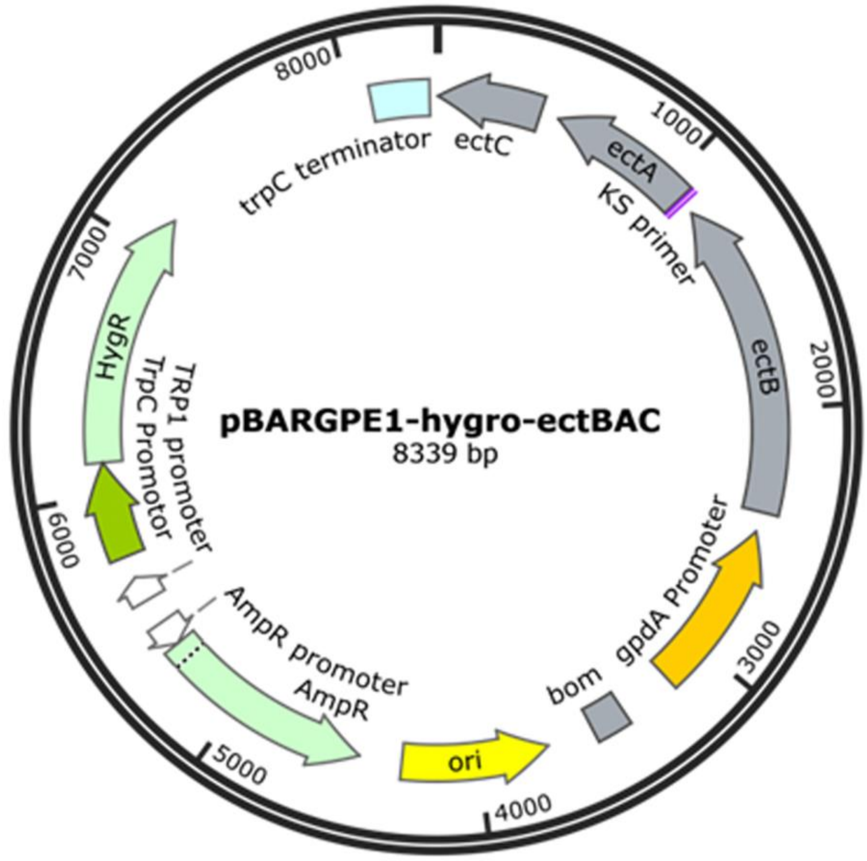

Figure S1. Plasmid map of pBARGPE1-*hygro-ectBAC*.

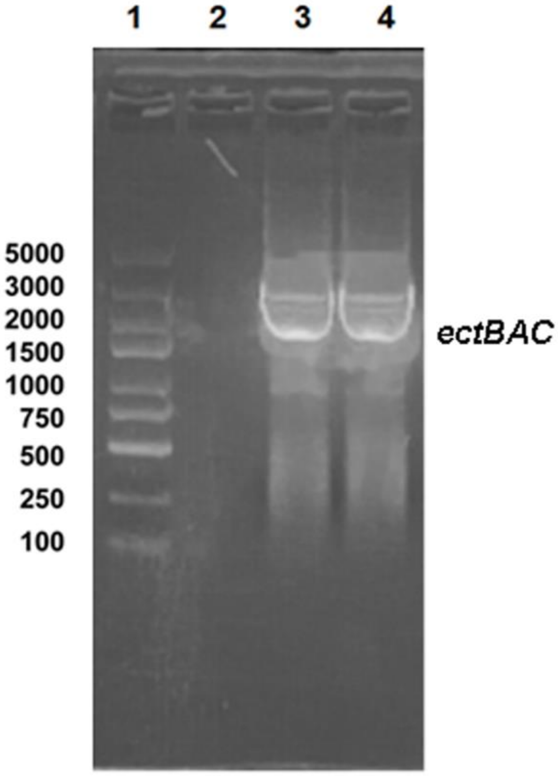

Figure S2. Diagnostic PCR of the expression of *ectBAC* gene cluster. Lane 1: DL5000 DNA ladder. Lane 2: blank. Lane 3&4: *ectBAC* fragment (~2000bp).

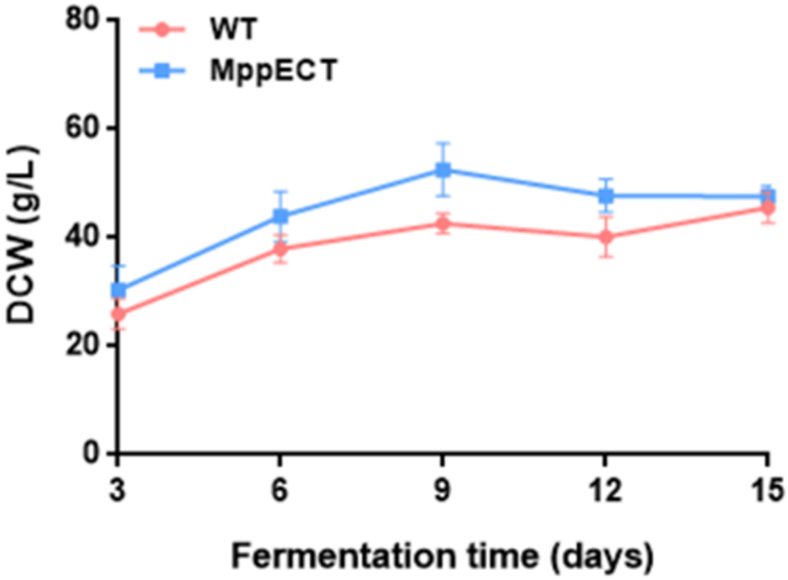

Figure S3. Dry cell weight (DCW) of *Monascus purpureus* ATCC 16365 WT and MppECT strains.

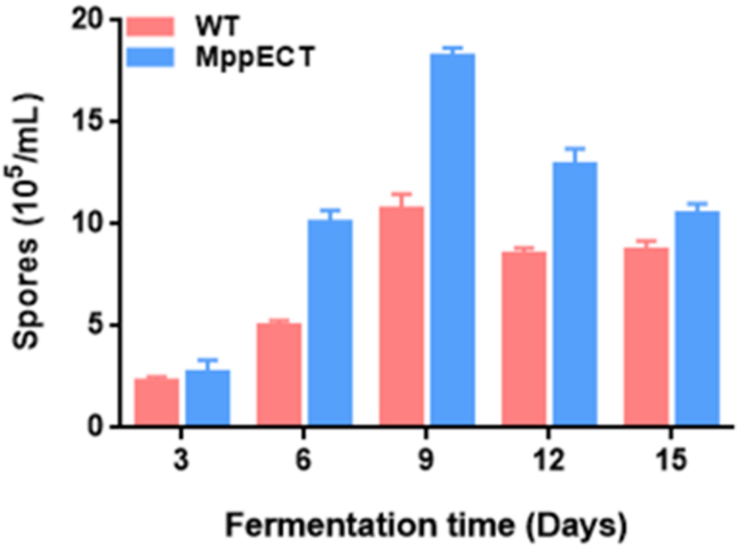

Figure S4. Spores number of *Monascus purpureus* ATCC 16365 WT and MppECT strains.

Table S1. Plasmids used in this study.

Plasmids	Description	source
pBARGPE1-*hygro*	Shuttle plasmid with gpdA and TrpC promoter, *hyg*$^{+}$, *amp*$^{+}$	Miaoling
pBARGPE1-*hygro-ectABC* (P1)	Pathway constructed in pBARGPE1-*hygro*	This study
pBARGPE1-*hygro-ectACB* (P2)	Pathway constructed in pBARGPE1-*hygro*	This study
pBARGPE1-*hygro-ectBAC* (P3)	Pathway constructed in pBARGPE1-*hygro*	This study
pBARGPE1-*hygro-ectBCA* (P4)	Pathway constructed in pBARGPE1-*hygro*	This study
pBARGPE1-*hygro-ectCAB* (P5)	Pathway constructed in pBARGPE1-*hygro*	This study
pBARGPE1-*hygro-ectCBA* (P6)	Pathway constructed in pBARGPE1-*hygro*	This study

Table S2 Primers used in this study.

Primers	Sequence (5′-3′)
vector-F	GGATCCTCTAGAGTCGAGGT
vector-R	GGTACCGCCCCGTCCGGTCC
ectABC-F (for P1)	GGACCGGACGGGGCGGTACCTTACAGCGGCTTCTGGTCGT
ectABC-R (for P1)	ACCTCGACTCTAGAGGATCCATGAACGCAACCACAGAGCC
ectACB-F (for P2)	GGACCGGACGGGGCGGTACCTCAGCTAAAGGCCTGCTTGG
ectACB-R (for P2)	ACCTCGACTCTAGAGGATCCATGAACGCAACCACAGAGCC
ectBAC-F (for P3)	GGACCGGACGGGGCGGTACCTTACAGCGGCTTCTGGTCGT
ectBAC-R (for P3)	ACCTCGACTCTAGAGGATCCATGCAGACCCAGATTCTCGA
ectBCA-F (for P4)	GGACCGGACGGGGCGGTACCTCAGATCTGGTCGGTCTGGA
ectBCA-R (for P4)	ACCTCGACTCTAGAGGATCCATGCAGACCCAGATTCTCGA
ectCAB-F (for P5)	GGACCGGACGGGGCGGTACCTCAGCTAAAGGCCTGCTTGG
ectCAB-R (for P5)	CCGTCGACCTCGACTCTAGAATGATCGTTCGCAATCTCGA
ectCBA-F (for P6)	GGACCGGACGGGGCGGTACCTCAGATCTGGTCGGTCTGGA
ectCBA-R (for P6)	CCGTCGACCTCGACTCTAGAATGATCGTTCGCAATCTCGA

Table S3 Sequence of gene cluster after codon optimization.

Gene name	DNA sequence after codon optimization
ectBAC	ATGCAGACCCAGATTCTCGAACGCATGGAGTCCGACGTTCGGACCT<br>ACTCCCGCTCCTTCCCGGTCGTCTTCACCAAGGCGCGCAATGCCCG<br>CCTGACCGACGAGGAAGGGCGCGAGTACATCGACTTCCTGGCCGG<br>TGCCGGCACCCTGAACTACGGCCACAACAACCCGCACCTCAAGCA<br>GGCGCTGCTCGACTATATCGACAGCGACGGCATCGTCCACGGCCTG<br>GACTTCTGGACTGCGGCCAAGCGCGACTATCTGGAAACCCTGGAA<br>GAGGTGATCCTCAAGCCGCGCGGTCTCGACTACAAGGTGCATCTGC<br>CCGGACCGACTGGCACCAACGCCGTCGAGGCGGCCATTCGCCTGG<br>CCCGGGTCGCCAAGGGGCGCCACAATATCGTCTCCTTCACCAACGG<br>CTTTCATGGCGTCACCATGGGCGCGCTGGCGACCACCGGTAACCGC<br>AAGTTCCGCGAGGCCACCGGTGGCGTGCCGACCCAGGCTGCTTCC<br>TTCATGCCGTTCGATGGCTACCTCGGCAGCAGCACCGACACCCTCG<br>ACTACTTCGAGAAGCTGCTCGGCGACAAGTCCGGCGGCCTGGACG<br>TGCCCGCGGCGGTGATCGTCGAGACAGTGCAGGGCGAGGGCGGTA<br>TCAATGTCGCCGGCCTGGAGTGGCTCAAGCGCCTCGAGAGCATCTG<br>CCGCGCCAATGACATCCTGCTGATCATCGACGACATCCAGGCGGGC<br>TGCGGCCGGACCGGCAAGTTCTTCAGCTTCGAGCATGCCGGCATCA<br>CGCCGGATATCGTGACCAACTCCAAGTCGCTGTCCGGTTACGGCCT<br>GCCGTTCGCTCACGTCCTGATGCGCCCCGAGCTCGACAAGTGGAA<br>GCCCGGTCAGTACAACGGCACCTTCCGCGGCTTCAACCTGGCTTTC<br>GCCACTGCTGCTGCCGCCATGCGCAAGTACTGGAGCGACGACACC<br>TTCGAGCGTGACGTGCAGCGCAAGGCTCGCATCGTCGAGGAACGC<br>TTCGGCAAGATCGCCGCCTGGCTGAGCGAGAACGGCATCGAGGCC<br>TCCGAGCGCGGCCGCGGGCTGATGCGGGGCATCGACGTGGGTTCC<br>GGCGATATCGCCGACAAGATCACCCACCAAGCCTTCGAGAACGGG<br>TTGATCATCGAAACCAGCGGTCAGGACGGCGAAGTGGTCAAGTGC<br>CTGTGCCCGCTGACCATTCCCGACGAAGACCTGGTCGAGGGACTC<br>GACATCCTCGAGACCAGCACCAAGCAGGCCTTTAGCTGAATCTTCA<br>GTATATTCATCTTCCCATCCAAGAACCTTTAATCAAGCTTATCGATAC<br>CGTCGACCTCGACTCTAGAATGAACGCAACCACAGAGCCCTTTAC<br>ACCCTCCGCCGACCTGGCCAAGCCCAGCGTGGCCGATGCCGTGGT<br>CGGCCATGAGGCCTCACCGCTCTTCATCCGCAAGCCAAGCCCCGAT<br>GACGGCTGGGGCATCTACGAGCTGGTCAAGTCCTGTCCGCCTCTCG<br>ACGTCAATTCCGCCTACGCCTATCTGTTGCTGGCCACCCAGTTCCGC<br>GATAGCTGCGCCGTGGCGACCAACGAAGAGGGCGAGATCGTCGGC<br>TTCGTTTCCGGCTACGTGAAGAGCAACGCCCCCGATACCTATTTCCT<br>CTGGCAGGTTGCCGTGGGCGAGAAGGCACGTGGCACCGGCCTGGC<br>CCGTCGTCTGGTGGAAGCCGTGATGACACGCCCGGAAATGGCCGA<br>GGTCCACCATCTCGAGACCACTATCACGCCCGACAACCAGGCGTC<br>CTGGGGCTTGTTCCGCCGTCTCGCCGATCGCTGGCAGGCGCCGTTG<br>AACAGCCGCGAATACTTCTCCACCGATCAACTCGGCGGTGAGCATG

ACCCGGAAAACCTCGTTCGCATCGGCCCGTTCCAGACCGACCAGA
TCTGAATCTTCAGTATATTCATCTTCCCATCCAAGAACCTTTAATCAA
GCTTATCGATACCGTCGACCTCGACTCTAGAATGATCGTTCGCAATC
TCGAAGAAGCGCGCCAGACCGACCGTCTGGTCACCGCCGAAAAC
GGCAACTGGGACAGCACCCGCCTGTCGCTGGCCGAAGATGGTGGC
AACTGCTCCTTCCACATCACCCGCATCTTCGAGGGTACCGAGACCC
ACATCCACTATAAGCATCACTTCGAGGCTGTTTATTGCATCGAAGGC
GAGGGCGAAGTGGAAACCCTGGCCGATGGCAAGATCTGGCCCATC
AAGCCGGGTGACATCTACATCCTCGACCAGCACGACGAGCACCTG
CTGCGCGCCAGCAAGACCATGCACCTGGCCTGCGTGTTCACGCCG
GGCCTGACCGGCAACGAAGTGCACCGCGAAGACGGTTCCTACGCA
CCTGCCGACGAAGCCGACGACCAGAAGCCGCTGTAA
